# Roles of Autophagy and Oxidative Stress in Cardiovascular Disease

**DOI:** 10.3390/antiox14101263

**Published:** 2025-10-20

**Authors:** Hyeong Rok Yun, Manish Kumar Singh, Sunhee Han, Jyotsna S. Ranbhise, Joohun Ha, Sung Soo Kim, Insug Kang

**Affiliations:** 1 Department of Biochemistry and Molecular Biology, School of Medicine, Kyung Hee University, Seoul 02447, Republic of Korea; foryou018@naver.com (H.R.Y.);; 2 Biomedical Science Institute, Kyung Hee University, Seoul 02447, Republic of Korea; 3 Department of Biomedical Science, Graduate School, Kyung Hee University, Seoul 02447, Republic of Korea

**Keywords:** autophagy, oxidative stress, cardiovascular disease

## Abstract

Autophagy and oxidative stress influence cardiovascular pathology. Autophagy mediates lysosome-dependent clearance of damaged proteins and organelles and maintains mitochondrial quality control, proteostasis, and metabolic flexibility. Reactive oxygen species (ROS) originate from mitochondrial respiration and enzymatic reactions during stress. At physiological levels, ROS function as redox signals that activate degradation and recycling, whereas excess oxidants damage lipids, proteins, and nucleic acids and promote cell loss. This review integrates evidence across cardiovascular disease, including atherosclerosis, ischemia reperfusion injury, pressure overload remodeling, heart failure, diabetic cardiomyopathy, arrhythmia, aging, and inflammation.

## 1. Introduction

Autophagy is a lysosome-dependent recycling program first described by Christian de Duve in 1963 and later defined at the genetic level when Yoshinori Ohsumi identified autophagy-related genes (ATGs) that are essential for autophagosome formation, providing fundamental insights into intracellular recycling in eukaryotic cells [1,2,3,4,5]. As a conserved catabolic pathway, autophagy sustains cellular homeostasis by routing damaged organelles and protein aggregates to lysosomal degradation and by coupling clearance to the recovery of metabolic building blocks [6,7,8,9]. As a result, autophagy maintains proteostasis, supports organelle quality control, and enables the remodeling that accompanies development and differentiation [3,10,11,12]. Autophagy is activated by a wide range of cellular stresses that include nutrient deprivation, such as loss of glucose or amino acids, oxidative and nitrosative stress, hypoxia, endoplasmic reticulum (ER) stress, mitochondrial injury, and exposure to toxic compounds [13,14]. Extensive experimental data demonstrate that autophagy maintains homeostasis under physiological load yet drives maladaptation when stress persists or autophagic flux is defective [15]. ROS are tightly linked to this pathway and act as upstream triggers, as well as downstream consequences, of autophagic activity [15,16,17]. In cardiovascular tissues, this autophagy–redox axis coordinates mitochondrial quality control with contractile and vascular function and shapes disease initiation and progression. Dysregulation of the autophagy–redox axis has been associated with metabolic disorders such as diabetes [18,19], malignancies [20], cardiovascular [21] and neurodegenerative diseases [22,23], immune dysregulation [24], and age-related decline [23,25].

Mitochondria are the principal source of ROS during aerobic metabolism, with prominent production at complex I through reverse electron transport (RET) and at the complex III Qo site [26,27,28,29,30]. Key representatives of ROS include superoxide (O_2_•^−^) and hydrogen peroxide (H_2_O_2_), both of which emerge from the incomplete four-electron reduction in molecular oxygen within the mitochondrial respiratory chain, with superoxide rapidly dismutated by superoxide dismutases (SODs) into H_2_O_2_ [30]. Among ROS, hydroxyl radicals (•OH) are essentially diffusion-controlled and can cause severe injury to DNA, lipids, and proteins when not properly regulated [31,32]. At physiological levels, ROS function as essential signaling molecules involved in processes such as cell proliferation [33], differentiation [34], innate immune activation [35], programmed cell death [36], Ca^2+^ signaling [37], redox regulation [38], stem cell maintenance [39], and autophagy modulation [40]. However, excessive ROS accumulation can result in oxidative damage to lipids, proteins, and DNA, contributing to disease development [41].

Physiological levels of ROS promote autophagy as an adaptive mechanism to maintain cellular homeostasis and mitigate stress-induced damage [42,43]. When redox signaling becomes dysregulated, autophagic flux is compromised, leading to increased cellular stress and accelerated disease development [40,44]. Although substantial progress has been made in elucidating the roles of redox signaling and autophagy as individual processes, the precise molecular circuitry that orchestrates their bidirectional regulation remains incompletely defined [45,46]. A comprehensive understanding of this interplay is critical, as perturbations in redox–autophagy balance are increasingly recognized as central drivers of diverse pathological states [47,48].

Autophagy interfaces with cellular energetics through mechanistic target of rapamycin complex 1 (mTORC1) and AMP-activated protein kinase (AMPK) and with antioxidant defense through nuclear factor erythroid 2-related factor 2 (NRF2) [49,50]. These links position autophagy as both a responder and a controller of redox biology in the heart and vasculature [46,48,51]. Circulating markers of autophagic activity and mitochondrial injury have been reported in humans, including extracellular vesicle-bound microtubule-associated protein light chain 3 (LC3) and sequestosome 1 (SQSTM1/p62) fragments, mitochondrial DNA, and oxylipid signatures [52,53,54].

This review synthesizes advances in ROS–autophagy regulation in cardiovascular biology, explains how this crosstalk shapes mitochondrial dynamics and phenotypes, and evaluates human measurement strategies and therapeutic approaches to inform clinical translation.

## 2. Mechanisms of Autophagy and Oxidative Stress

### 2.1. Autophagy Pathway and Selectivity

Eukaryotic cells use three lysosome-centered routes for intracellular clearance. These are macroautophagy, microautophagy, and chaperone-mediated autophagy (CMA). All three routes remove damaged or obsolete proteins and organelles and return their breakdown products to cellular metabolism [55,56].

Macroautophagy is the dominant route discussed in this review. The pathway begins with the formation of a phagophore, also called an isolation membrane. The phagophore expands to enclose selected material and becomes an autophagosome with a double membrane. The autophagosome then fuses with a lysosome to create an autolysosome, where cargo is degraded by lysosomal hydrolases. The resulting amino acids, fatty acids, and sugars are released into the cytosol and support synthesis and energy production. Although macroautophagy was once viewed as nonselective, it frequently operates in a selective mode through LC3 or GABARAP-interacting receptors that recognize long-lived proteins, aggregates, damaged mitochondria, or invading microbes [56,57].

Microautophagy delivers cytosolic components directly into the lysosomal lumen. Uptake occurs through invagination, protrusion, or septation of endosomal or lysosomal membranes. In mammalian cells, an endosomal variant packages cargo into intraluminal vesicles and depends on critical endosomal sorting complexes required for transport (ESCRT) machinery [58,59].

CMA is a selective route present in many mammalian tissues but absent from budding yeast. Cytosolic HSC70 recognizes substrates that contain KFERQ-like motifs and delivers them to lysosome-associated membrane protein 2 (LAMP2) on the lysosomal membrane. The substrate is unfolded and translocated across the membrane with the assistance of luminal chaperones and is then degraded [60].

In this review, we focus on macroautophagy, hereafter referred to as autophagy, with emphasis on the mechanism, regulation, and selectivity of autophagic cargo.

### 2.2. Core ATG Machinery and Regulatory Networks

**Figure 1:**
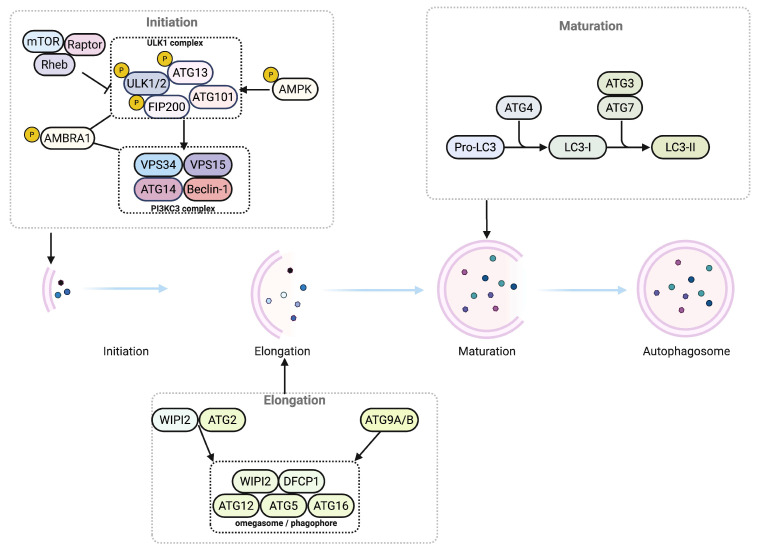
Core modules and sequence of macroautophagy. Macroautophagy progresses from initiation to elongation and maturation and culminates in a sealed autophagosome. In the initiation module, inhibition of mTORC1, together with AMPK activity, permits activation of the ULK1 complex (ULK1 or ULK2, ATG13, FIP200, ATG101). ULK1 and AMBRA1 engage the class III phosphatidylinositol 3 kinase complex (VPS34, VPS15 p150, beclin-1, ATG14), which generates phosphatidylinositol 3 phosphate on ER-derived omegasomes to recruit DFCP1 and WIPI2 and nucleate the phagophore. During elongation, ATG9A or ATG9B vesicles and ATG2 supply the membrane, while the ATG12–ATG5–ATG16L1 complex assembles on the outer phagophore surface and positions the LC3 lipidation machinery. In maturation, pro-LC3 is cleaved by ATG4 to LC3-I and conjugated to phosphatidylethanolamine by ATG7 and ATG3 to form LC3-II, which decorates the autophagosomal membrane until closure. (Figure created in http://biorender.com.)

Autophagy is regulated by an integrated signaling network. Yeast expresses about 40 ATG proteins, while mammals retain roughly 20 core ATG proteins that drive the pathway [56,61]. Nutrient- and energy-sensing kinases, chiefly mTORC1 and AMPK, modulate ATG activity and govern induction and flux [62]. The process is activated by nutrient withdrawal, hypoxia, oxidative stress, ER stress, and infection [63]. During nutrient sufficiency, mTORC1 phosphorylates the uncoordinated-51-like kinase 1 (ULK1)–ATG13–focal adhesion kinase family-interacting protein of 200 kDa (FIP200)–ATG101 complex and blocks autophagy initiation [64]. Metabolic stress activates AMPK, which inhibits mTORC1 by targeting Rheb and regulatory-associated protein of mTOR (RAPTOR) and simultaneously phosphorylates ULK1 at Ser317 and Ser777, thereby activating the kinase [64]. Recent work shows that ULK1 is palmitoylated by the acyltransferase zinc finger DHHC-type palmitoyltransferase 13 (ZDHHC13), which increases ULK1 membrane association and strengthens initiation [65]. Recent structural work resolved a complex comprising the ULK1–ATG13–FIP200–ATG101 complex and the class III PI3KC3-C1 (ATG14L–VPS34–VPS15–beclin-1), clarifying how ULK1 activation is physically coupled to VPS34 lipid kinase activity at the earliest step of autophagosome formation [66]. Active ULK1 phosphorylates beclin-1 and beclin-1-regulated autophagy protein 1 (AMBRA1), recruiting the class III phosphatidylinositol 3 kinase complex with VPS34, ATG14L, VPS15, and beclin-1 to the phagophore assembly site, where phosphatidylinositol 3-phosphate is generated [67]. Double FYVE domain-containing protein 1 (DFCP1) is now recognized as a PI3P effector with ATPase activity that constricts omegasomes and accelerates their transition into growing phagophores [68,69]. PI3P-binding proteins such as WD repeat domain phosphoinositide-interacting protein 2 (WIPI2) and DFCP1 recognize this lipid and initiate phagophore nucleation and membrane remodeling [67]. Although the ER supplies most membrane material, the Golgi, endosomes, mitochondria, and plasma membrane also contribute. PI3KC3 activity is enhanced by UV radiation resistance-associated gene (UVRAG), inhibited by RUN domain- and cysteine-rich domain-containing beclin-1-interacting protein (Rubicon), and beclin-1 is sequestered by phosphorylated B-cell lymphoma 2 (BCL-2) family proteins under basal conditions [70]. Phagophore expansion relies on two ubiquitin-like conjugation cascades. First, ATG12 conjugates to ATG5 and, together with ATG16L1, assembles on the outer membrane and functions as an E3-like enzyme for LC3 lipidation [71,72]. Second, ATG4 cleaves pro-LC3 to expose a C-terminal glycine, and ATG7 and ATG3 then attach phosphatidylethanolamine to produce LC3-II [73]. Lipidated LC3 embeds in both phagophore leaflets and serves as a molecular tag for selective cargo capture by adaptor proteins, such as p62, nuclear dot protein 52 kDa (NDP52), neighbor of BRCA1 gene 1 (NBR1), and optineurin (OPTN), all of which bind LC3 through LC3-interacting regions [74,75]. The ATG2–WIPI complex shuttles lipids from the ER to the growing phagophore and supports membrane elongation [76,77]. ATG9A functions as a trimeric lipid scramblase, and its tissue-restricted paralog ATG9B also forms a homotrimeric lipid scramblase that may partially compensate for ATG9A in certain contexts of autophagosome biogenesis [78]. ATG9, the only transmembrane ATG protein, adds further bilayer material by trafficking vesicles between the Golgi apparatus and the phagophore assembly site, and ULK1-dependent phosphorylation of ATG9 at Ser14 enhances its recruitment [79,80]. As elongation proceeds, the endosomal sorting complex required for transport closes the edges of the double membrane to form a sealed autophagosome. The VPS4 ATPase is an ESCRT cofactor for this scission step and for the orderly handoff to downstream maturation [81,82]. Gamma-aminobutyric acid receptor-associated protein (GABARAP) paralogs, functionally analogous to LC3, cooperate in membrane elongation and cargo capture and complement the LC3 conjugation system [83]. Once closure is complete, the ATG12–ATG5–ATG16L1 scaffold dissociates, and ATG4 removes LC3-II from the outer membrane for recycling [84,85]. The mature autophagosome then travels along microtubules to the perinuclear region and fuses with lysosomes through soluble N-ethylmaleimide-sensitive factor attachment protein receptor (SNARE) pairs [86,87]. In mammalian cells, syntaxin 17 (STX17) on the autophagosome pairs with SNAP29 and vesicle-associated membrane protein 8 (VAMP8) on the lysosome as a predominant fusion axis, whereas, under specific conditions, an alternative pathway involving STX17–SNAP47–VAMP7/VAMP8 or a YKT6–SNAP29–STX7 route can mediate autophagosome–lysosome fusion, with SNAP47 contributing to homotypic fusion and vacuole protein sorting (HOPS) recruitment [70,88,89,90,91]. This redundancy adds resilience to the final fusion step. Within the autolysosome, lysosomal hydrolases such as cathepsins and acid lipases degrade the inner membrane and cargo to amino acids, fatty acids, and sugars, which exit through permeases for biosynthesis and ATP production [92,93]. Prolonged starvation drives transcription factor EB (TFEB) into the nucleus, where it upregulates autophagy and lysosome-related genes and increases degradative capacity [94]. Autophagy also operates in a selective mode. LIR-containing adaptors direct LC3 or GABARAP to specific substrates and enable mitophagy through PTEN-induced kinase 1 (PINK1) and Parkin, lipophagy, and xenophagy [74,95,96]. These functions are essential for quality control, immunity, and metabolic balance [5]. Defects in these pathways contribute to cancer, neurodegeneration, metabolic disease, and cardiovascular disorders and highlight autophagy as an important therapeutic target [97,98,99]. Taken together, the core autophagy machinery comprises four cooperating modules (initiation, nucleation, elongation with conjugation, and closure with fusion) that are directly regulated by energetic and redox signals. Benefits require completion of autophagic flux through lysosomal fusion. An overview of the autophagy pathway is shown in Figure 1.

### 2.3. Molecular Biology of Oxidative Stress

Mitochondria synthesize adenosine triphosphate (ATP) and constitute the principal intracellular source of ROS. Although several pathways generate ROS, the mitochondrial electron transport chain (mETC) is the dominant site of formation during aerobic metabolism [30,100]. Throughout oxidative phosphorylation, electrons traverse four multi-subunit complexes (I–IV). Electron leakage from complex I (NADH: ubiquinone oxidoreductase) and complex III (ubiquinol: cytochrome c oxidoreductase) partially reduces molecular oxygen, generating superoxide (O_2_•^−^), which is subsequently dismutated into hydrogen peroxide (H_2_O_2_) [101,102]. Principal mitochondrial ROS (mtROS) consist of superoxide, hydrogen peroxide, hydroxyl radical (•OH), and singlet oxygen (^1^O_2_). Under physiological conditions, intracellular ROS levels are tightly controlled and remain low [41,103]. Protection against ROS-induced injury relies on an integrated antioxidant network containing enzymatic catalysts and non-enzymatic scavengers [104]. Mitochondrial superoxide is rapidly converted into hydrogen peroxide by superoxide dismutases: Cu/ZnSOD (SOD1) in the intermembrane space and cytosol, and MnSOD (SOD2) in the matrix [105,106]. Although less reactive than superoxide, H_2_O_2_ reacts with ferrous iron via Fenton chemistry to produce highly cytotoxic hydroxyl radicals [107]. Protonation of superoxide yields the hydroperoxyl radical (HOO•), which initiates lipid peroxidation of polyunsaturated fatty acids in mitochondrial membranes [108]. Endogenous mitochondrial nitric oxide (NO) reacts with superoxide to generate peroxynitrite (ONOO^−^), a potent oxidant species that promotes protein tyrosine nitration—commonly detected as 3-nitrotyrosine—and drives thiol oxidation [109,110,111]. By contrast, S-nitrosylation typically arises from NO-derived nitrosating equivalents, such as dinitrogen trioxide (N_2_O_3_) or via transnitrosation reactions, rather than from peroxynitrite [112,113].

**Figure 2:**
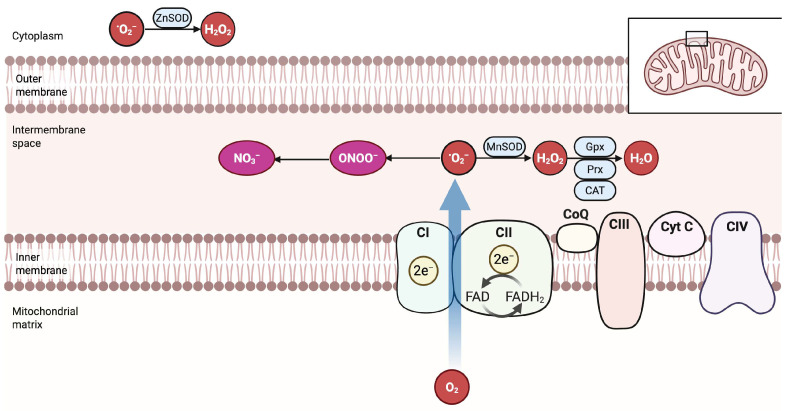
Compartmental handling of mtROS. Electron transfer through complexes I and II feeds the respiratory chain and gives rise to superoxide O_2_•^−^ at defined sites. In the matrix, MnSOD converts superoxide into hydrogen peroxide (H_2_O_2_), while Cu/ZnSOD in the intermembrane space and cytoplasm performs the same reaction. H_2_O_2_ diffuses across compartments and is reduced to water by glutathione peroxidases, peroxiredoxins, and catalase. Superoxide (O_2_•^−^) reacts with nitric oxide to form peroxynitrite (ONOO^−^), which yields secondary oxidants and terminates as nitrate (NO_3_^−^). The scheme also locates CoQ, complex III, cytochrome c, and complex IV to relate oxidant formation to the architecture of the inner membrane. (Figure created in http://biorender.com.)

Hydrogen peroxide is detoxified by peroxiredoxins (Prx3 and Prx5), glutathione peroxidases (GPx1, GPx2, and GPx4), thioredoxin 2 (Trx2), and catalase [114,115,116,117,118]. This enzyme set requires reducing equivalents supplied chiefly by glutathione (GSH) and the thioredoxin cycle. During detoxification, GSH is oxidized into glutathione disulfide (GSSG) and regenerated by glutathione reductase (GR), whereas oxidized Trx2 is restored by NADPH-dependent thioredoxin reductase (TrxR) [117,119]. Matrix NADPH is replenished by isocitrate dehydrogenase (IDH), malate dehydrogenase (MDH), and nicotinamide nucleotide transhydrogenase (NNT), maintaining the reductive environment necessary for antioxidant function [120,121,122]. Catalase further decomposes hydrogen peroxide into water and molecular oxygen, adding an additional layer of protection. Collectively, this multilayered defense mitigates mitochondrial oxidative stress and prevents ROS-mediated cellular injury [26]. Beyond cytotoxicity, mtROS act as signaling molecules that regulate autophagy [123]. Nutrient deprivation—such as withdrawal of glucose, amino acids, or serum—elevates mtROS levels and activates autophagy-related pathways [124]. Oxidative stress-induced autophagy limits apoptotic cell death and confers cytoprotection [125]. In contrast, defective autophagic flux promotes ROS accumulation and exacerbates oxidative injury, whereas pharmacological antioxidants can blunt or abolish the autophagic response [126]. The evidence, therefore, supports bidirectional crosstalk in which mtROS both initiate and modulate autophagic signaling. Redox control modifies autophagy at multiple layers that include ATG4 oxidation, ULK1 and beclin-1 phosphorylation, and TFEB nuclear translocation through lysosomal Ca^2+^ release [124,127,128]. Spatial organization is critical because mitochondria–ER contact sites scaffold autophagosome initiation and regulate Ca^2+^ and lipid exchange, which tunes both mitophagy and metabolic output [129,130]. Failure of flux increases cytosolic mitochondrial DNA (mtDNA) and sustains cGAS–STING signaling, linking redox imbalance to innate immune activation [131,132]. The generation of mtROS is schematized in Figure 2.

## 3. Relationship Between Autophagy and Oxidative Stress in Cardiovascular Disease

**Figure 3:**
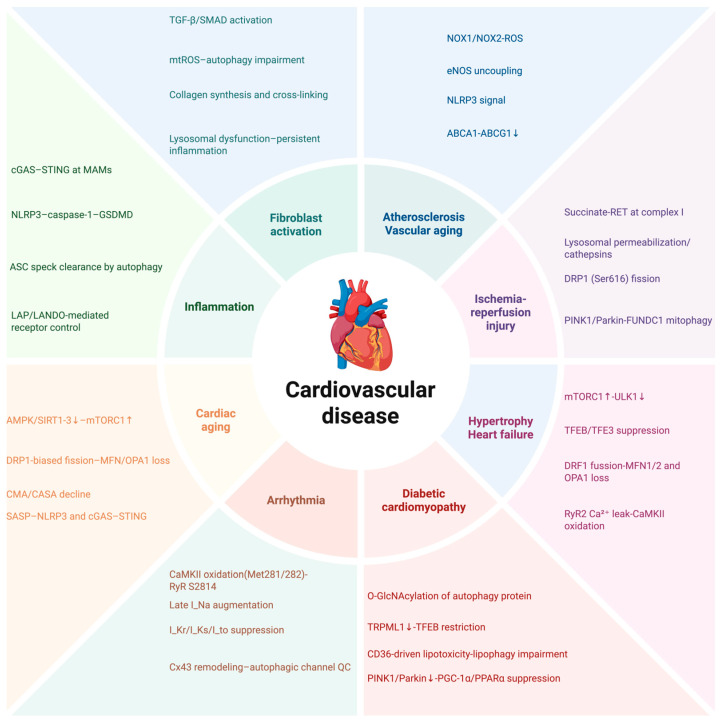
Central schematic of autophagy–redox coupling across cardiovascular disease. Eight disease modules are illustrated, each annotated with four representative signaling nodes. Examples include NOX1/NOX2-derived ROS and eNOS uncoupling (atherosclerosis and vascular aging); succinate-driven reverse electron transport and DRP1-mediated fission (ischemia–reperfusion injury); mTORC1 ↑ with ULK1 ↓ (hypertrophy and heart failure); O-GlcNAcylation and reduced TRPML1 (diabetic cardiomyopathy); CaMKII oxidation with augmented late I_Na (arrhythmia); decreased AMPK/SIRT signaling with increased mTORC1 activity (cardiac aging); cGAS–STING and NLRP3 activation (inflammation); and TGF-β/SMAD signaling (fibroblast activation). Shared therapeutic entry points are indicated. Abbreviations are defined in the text. (Figure created in http://biorender.com.)

Autophagy and oxidative stress exhibit bidirectional crosstalk in cardiovascular tissues. Within the cardiovascular system, autophagy and mitophagy interface tightly with redox signaling to preserve protein and organelle quality control and metabolic homeostasis across cardiomyocytes [133], endothelial cells [134], vascular smooth muscle cells [135], and immune cells [12]. Autophagy maintains the integrity of the contractile apparatus, stabilizes Ca^2+^ handling and mitochondrial bioenergetics, and attenuates transient oxidative challenges by clearing damaged proteins and organelles and sustaining lysosomal competence [136,137]. In contrast, sustained oxidative stress coupled with impaired autophagic flux promotes mitochondrial dysfunction [138], proteotoxic stress [139], inflammatory amplification [140], and maladaptive remodeling [141]. Whether their interaction is protective or harmful depends on the identity and magnitude of the stimulus, cell type, disease stage, and activation duration, spanning outcomes from cytoprotective adaptation to pathological injury [142]. An overview of cardiovascular disease is shown in Figure 3. Key signaling modules include cascades of the AMPK/mTOR/ULK1 and PINK1/Parkin pathway, together with redox-sensitive transcriptional programs such as NRF2 [54,143,144]. Collectively, these systems integrate redox and autophagy signals to coordinate organelle quality control and metabolic substrate utilization, thereby shaping disease phenotypes that range from ischemia–reperfusion injury and diabetic cardiomyopathy to atherosclerosis and heart failure. Evidence summarized in this section was identified through structured searches and was limited to peer-reviewed studies published from January 2010 through October 2025. Inclusion required the use of human cardiovascular cells or tissues; explicit interrogation of autophagy, mitophagy, and redox pathways using defined genetic or pharmacologic perturbations; at least one flux measurement such as LC3 turnover, p62 degradation, or a validated mitophagy reporter; and quantitative assessment of oxidative stress. For pharmacologic investigations, eligibility required evidence of target engagement, such as AMPK activation, mTOR modulation, TFEB nuclear translocation, or PINK1/Parkin pathway activity. For human studies, eligibility required pre-specified clinical or imaging endpoints, and studies that employed validated circulating biomarkers of autophagy, mitophagy, or redox status were also considered. Key terms and representative measurement approaches are summarized in Table 1.

### 3.1. Atherosclerosis and Vascular Aging

Atherosclerosis and vascular aging accelerate when oxidative stress exceeds autophagic quality control in the vascular wall. Oxidized lipids and excessive ROS drive endothelial NO synthase (eNOS) uncoupling, diminish nitric oxide availability, and activate NF-κB-dependent inflammatory signaling [145,146]. Across the vascular wall, the dominant sources of ROS differ by cell type. In the endothelium and smooth muscle, NADPH oxidases (NOX1/NOX2) and eNOS uncoupling predominate. In macrophages, mitochondrial-derived and NOX-derived ROS integrate with lipotoxic signaling and promote inflammasome activation [147,148,149]. Loss of autophagic competence leads to p62 accumulation and lysosomal dysfunction; facilitates the nucleotide oligomerization domain (NOD)-like receptor (NLR) family of proteins 3 (NLRP3) inflammasome in response to cholesterol crystals; and impairs apoptotic cell clearance and lipid processing in macrophages, thereby accelerating the buildup of lipid-accumulating macrophages and promoting features of plaque instability [150,151]. By contrast, physiological mitophagy mediated by PINK1/Parkin and hypoxia-responsive receptors such as BCL2/adenovirus E1B 19 kDa-interacting protein 3 (BNIP3), NIX (Bcl-2/adenovirus E1B 19 kDa-interacting protein 3, long form; BNIP3L), and FUN14 domain-containing 1 (FUNDC1) limits mtDNA release [131]. Autophagy also supports macrophage lipophagy and cholesterol efflux via ATP-binding cassette transporters A1 (ABCA1) and G1 (ABCG1), collectively improving lipid homeostasis within lesions [152]. In vascular smooth muscle cells, autophagy limits osteogenic differentiation by attenuating Runt-related transcription factors 2 (RUNX2) and SRY-box transcription factor 9 (SOX9) programs; reduces the biogenesis of calcifying extracellular vesicles; and maintains the contractile phenotype through preservation of myocardin and SRF while preventing maladaptive Krüppel-like factor 4 (KLF4)-driven switching, thereby slowing medial calcification and vascular stiffening [153,154,155]. With aging, autophagy and mitophagy decline across the vascular wall, leading to accumulation of dysfunctional mitochondria, mtDNA damage, and chronic inflammation that amplify elastin fragmentation, collagen deposition, and endothelial-to-mesenchymal transition, together culminating in accelerated atherogenesis and vascular aging [156,157]. Hemodynamic shear induces KLF2 and KLF4 signaling that promotes nitric oxide signaling and anti-inflammatory gene expression in the endothelium [158]. Autophagy supports this state by maintaining eNOS coupling and by limiting mtROS [159]. In human plaques, reduced autophagy markers correlate with increased necrotic core and calcification, supporting a link between defective quality control and lesion complexity [160,161]. In addition, microRNAs are implicated in these pathways. The miR-17-92 cluster, including miR-17-5p, modulates autophagy, and miR-92a associates with coronary plaque development and transition to unstable plaque [162,163]. Clonal hematopoiesis with loss-of-function variants in Tet methylcytosine dioxygenase 2 (TET2) or DNA (cytosine-5)-methyltransferase 3A (DNMT3A) increases systemic inflammatory tone and accelerates atherogenesis [5,164,165]. Autophagy modulates this axis by limiting mitochondrial DNA leakage and curbing NLRP3 and cGAS–STING signaling in monocyte–macrophage lineages, thereby attenuating the inflammatory amplification linked to clonal hematopoiesis [131,132,166]. Human imaging and proteomic studies that couple plaque phenotype with autophagy signatures would clarify whether impaired quality control marks lesions at the highest clinical risk [161,167]. Clinically, the redox and inflammation axis has been targeted, for example, interleukin 1β blockade with canakinumab in CANTOS and colchicine therapy in COLCOT and LoDoCo2, and human plaques exhibit autophagy pathway alterations, with CMA acting protectively, underscoring the translational relevance of autophagy and redox coupling in atherothrombosis [168,169,170]. Co-targeting autophagy and oxidative stress may slow atherosclerotic plaque progression and requires prospective validation in clinical trials using biomarkers. Therapeutically, plaque stabilization may be achieved by integrating NOX inhibition or eNOS recoupling, targeted activation of autophagy/mitophagy, and inflammasome blockade with biomarker-guided trials to validate benefits.

### 3.2. Ischemia–Reperfusion (I/R) Injury

I/R consists of two distinct stresses that differentially regulate autophagy. During ischemia, ATP depletion and acidosis activate AMPK and inhibit mTORC1, engaging ULK1 and the beclin-1–VPS34 complex to initiate adaptive autophagic signaling that removes damaged organelles, recycles substrates, and helps maintain viability [171,172]. Reperfusion, particularly during the initial minutes, exposes the myocardium to pronounced oxidative and Ca^2+^ loads driven by succinate oxidation-induced reverse electron transport at complex I, together with NADPH oxidase and xanthine oxidase activity, along with Ca^2+^ influx and mitochondrial uptake via the mitochondrial calcium uniporter (MCU) [173,174,175,176,177]. The resulting redox and Ca^2+^ stress amplifies autophagic activity, destabilizes lysosomes, and induces lysosomal membrane permeabilization with cathepsin release, culminating in mitochondrial permeability transition (MPT)-dependent regulated necrosis [175,178]. Autophagy and mitophagy during ischemia are generally protective by limiting protein aggregation, clearing dysfunctional mitochondria, and preserving metabolic flexibility. In contrast, sustained activation during late reperfusion or impaired autophagosome clearance is deleterious. It depletes amino acid pools, compromises lysosomal integrity, and impairs contractile recovery [179,180,181]. Reinforcement of mitophagy through PINK1/Parkin recruitment to depolarized mitochondria or FUNDC1 dephosphorylation under hypoxia inhibits mtROS, stabilizes the mitochondrial membrane potential (ΔΨm), and improves energetic balance [21,182,183]. In contrast, Dynamin-related protein 1 (DRP1) hyperactivation with Ser616 phosphorylation and BNIP3-mediated outer-membrane permeabilization promote mitochondrial fragmentation, sensitize mitochondria to MPT, and increase cell loss [184]. Endothelial and immune cells influence outcomes. MicroRNAs that regulate autophagy, including members of the miR-17-92 cluster, modulate redox-responsive signaling during I/R and may influence the extent of myocardial injury and recovery [96,162]. When autophagic flux is impaired, microvascular obstruction increases, and inflammation is amplified in the reperfused myocardium [185,186]. Pharmacologic strategies that activate AMPK, temper mTORC1, stabilize lysosomes, attenuate pathological fission, or enhance PINK1/Parkin and FUNDC1 signaling show cardioprotection in preclinical models, positioning autophagy–mitophagy and redox control as actionable targets in I/R injury [171,178]. Reperfusion injury reflects succinate accumulation during ischemia and rapid oxidation on reflow that drives reverse electron transport at complex I [173,176,177]. Autophagy modulates reperfusion injury at two levels by stabilizing lysosomes to limit cathepsin release and by restoring mitophagy to remove sources of reverse electron transport [21,178]. Autophagy modulates ferroptosis and necroptosis during reperfusion injury [187]. Lipid peroxidation and iron handling determine whether autophagy remains protective or transitions to cell death-permissive states [188]. In patients, cardiac MRI markers of microvascular obstruction and intramyocardial hemorrhage predict adverse outcomes after reperfusion injury [189]. The succinate-driven reverse electron transport mechanism is supported by human and organ data [190,191]. The mitochondria-targeted peptide elamipretide was neutral in the EMBRACE STEMI pilot, which highlights the gap between mechanism and efficacy [187,192,193]. Ischemia activates protective autophagy, whereas oxidative stress during reperfusion impairs autophagic fusion, leading to defective clearance and myocardial injury. Accordingly, early I/R cardioprotection should combine AMPK activation and mTORC1 inhibition with lysosome stabilization, DRP1 inhibition, and mitigation of succinate-driven reverse electron transport, while restoring PINK1–Parkin or FUNDC1-mediated mitophagy.

### 3.3. Hypertrophic Cardiomyopathy (HCM) and Heart Failure (HF)

HCM and HF are characterized by altered autophagy and lysosomal dysfunction in cardiomyocytes. Pressure overload activates mTORC1 and suppresses ULK1, increasing protein synthesis [64,127,194]. Early activation of the AMPK/ULK1 axis is associated with adaptive remodeling through selective clearance of damaged organelles and preserving lysosomal function [195,196,197]. As HF progresses, lysosomal acidification decreases; TFEB-driven lysosomal and autophagy genes are suppressed; and core ATG proteins acquire inhibitory post-translational modifications, including O-GlcNAcylation and oxidative or nitrosative modifications such as carbonylation, S-nitrosylation, and S-glutathionylation, culminating in impaired autophagic flux [36,198]. Impaired v-ATPase activity and TRPML1-dependent lysosomal Ca^2+^ release attenuate TFEB/TFE3 activation [199]. Concomitantly, excess DRP1-dependent fission with reduced mitofusin 1/2 (MFN1/2) and Optic Atrophy 1 (OPA1) disrupts cristae and mitophagy, thereby increasing mtROS and mtDNA damage [47,200,201]. In cardiomyocytes, the ubiquitin–proteasome system (UPS) and chaperone-assisted selective autophagy (CASA) are essential for proteostasis, and their failure promotes decompensation [139]. Oxidative and nitrosative post-translational modifications of sarcomeric proteins and chaperones increase CASA levels and proteasomal clearance, reinforcing proteotoxic stress in HF [139,202]. In parallel, metabolic reprogramming, with reduced fatty acid oxidation, impaired Peroxisome proliferator-activated receptor gamma coactivator 1-alpha (PGC-1α)–PPARα (Peroxisome proliferator-activated receptorα) signaling, and imbalance across the sirtuin (SIRT)–AMPK-mTOR axis, exacerbates the mismatch between ATP demand and supply [203]. Redox modifications of ryanodine receptor 2 (RyR2) increase sarcoplasmic reticulum Ca^2+^ leak, while microtubule-dependent trafficking defects and loss of mitochondria–sarcomere coupling further impair excitation–contraction coupling [202,204]. HF with preserved ejection fraction (HFpEF) shows systemic inflammation, endothelial dysfunction, and microvascular rarefaction with reductions, distinct from HF reduced ejection fraction (HFrEF) [133,205]. In HFpEF, sex-based differences in autophagy and mitochondrial signaling influence microvascular inflammation, stiffness, and diastolic function and, therefore, merit sex-stratified analyses in clinical studies. Therapeutic decisions should be guided by metabolic and inflammatory profiles. Human genetic evidence links impaired autophagy to cardiomyopathy [206,207]. Danon disease (glycogen storage disease Type IIB) with LAMP2 deficiency produces severe hypertrophic or dilated phenotypes with lysosomal dysfunction [208,209,210]. Bcl2-associated athanogene (BAG3) variants disrupt CASA and are associated with myofibrillar myopathy and dilated cardiomyopathy [139,211]. Accordingly, management of HCM and HF should integrate mTOR inhibition or AMPK activation with TFEB-directed lysosomal restoration and mitophagy enhancers, complemented by RyR2 or CaMKII control and suppression of late sodium current to stabilize excitation–contraction coupling.

### 3.4. Diabetic Cardiomyopathy (DCM) and Metabolic Syndrome

Hyperglycemia, excess fatty acids, and insulin resistance result in mitochondrial and NADPH oxidase-derived ROS, advanced glycation end products (AGEs), and lipid peroxidation species that aggravate ER stress and suppress both the initiation and completion of autophagy through ULK1, the beclin-1–VPS34 complex, and LC3 processing [212,213]. In the diabetic heart, increased flux through the hexosamine pathway elevates O-GlcNAcylation on key autophagy proteins and lysosomal regulators, which suppresses induction and impairs autophagic flux, while p62 accumulation with Kelch-like ECH-associated protein 1 (KEAP1) sequestration produces self-reinforcing NRF2 activation that reprograms redox and lipid pathways [214,215,216]. Defective lysosomal acidification, reduced v-ATPase activity, and impaired lysosomal Ca^2+^ release via transient receptor potential mucolipin 1 (TRPML1) limit TFEB/TFE3 nuclear translocation and diminish degradative capacity [94,217,218]. Mitophagy signaling is frequently attenuated, with reduced PINK1 stabilization and Parkin recruitment to depolarized mitochondria, altered BNIP3/NIX activity, and an imbalance in mitochondrial dynamics marked by DRP1-dependent fission with loss of MFN1/2 and OPA1 [47]. Attenuated mitophagy and altered dynamics elevate mtROS, disrupt cristae structure, depress oxidative phosphorylation, and diminish respiratory reserve [219,220]. In parallel, impaired lipophagy and dysregulated CD36-mediated uptake drive accumulation of toxic lipid intermediates such as ceramides and diacylglycerols that activate protein kinase C (PKC) signaling and impair the insulin receptor substrate (IRS)/PI3K/AKT signaling, whereas suppression of PGC-1α/PPARα compromises fatty acid oxidation and shifts substrate utilization toward metabolically inefficient pathways [221,222]. Ca^2+^ handling deteriorates through RyR2 oxidation, sarcoplasmic reticulum Ca^2+^ATPase 2a (SERCA2a) O-GlcNAcylation and acetylation, and altered mitochondria–SR junctional microdomains, which together aggravate diastolic stiffness and impair excitation–contraction coupling [213,223]. At the tissue level, eNOS uncoupling and microvascular rarefaction reduce perfusion and amplify inflammation [224]. Autophagy defects in macrophage and fibroblast promote NLRP3 activation and transforming growth factor β (TGF-β)-driven matrix deposition, accelerating interstitial fibrosis and ventricular remodeling [225]. Exercise, metformin, and caloric restriction enhance AMPK activity and reactivate SIRT3–PGC-1α signaling, thereby enhancing mitophagy and mitochondrial biogenesis, improving lysosomal function, and normalizing fatty acid oxidation and redox balance [226,227]. Sodium–glucose cotransporter-2 (SGLT2) inhibitors, such as empagliflozin, attenuate oxidative stress, modulate Na^+^/H^+^ exchange and cytosolic Na^+^ handling, and improve autophagy–lysosome function, leading to improved energetic efficiency and structural remodeling in diabetic hearts [228,229,230]. Ketone oxidation and anaplerotic flux influence autophagy and mitophagy through changes in NADH and NADPH balance. SGLT2 inhibition increases ketone availability and improves lysosomal function and mitochondrial quality control in experimental systems, contributing to improved diastolic function, independent of glucose control [231,232]. Peroxisomal β-oxidation and aldehyde detoxification influence redox homeostasis in diabetic hearts. Reduced aldehyde dehydrogenase activity increases reactive aldehydes that modify mitochondrial proteins and suppress respiration. Enhancing mitochondrial biogenesis, together with improved lysosomal function, optimizes substrate use and improves respiratory capacity and reduces accumulation of ceramides and diacylglycerols that impair insulin signaling [233,234,235]. Hence, DCM is a tractable target for SGLT2-based metabolic therapy plus AMPK or SIRT3 activation to enhance TFEB-lysosome function and mitophagy, normalize fatty acid oxidation and ketone oxidation, and limit inflammatory remodeling.

### 3.5. Arrhythmia and Electrophysiology

Oxidative stress disrupts Ca^2+^ handling, mitochondrial signaling, and ion channel function in atrial and ventricular arrhythmias. ROS oxidize Ca^2+^/calmodulin-dependent protein kinase II (CaMKII) at Met281/282, generating calmodulin-independent activity that increases phosphorylation of RyR2 at S2814, enhances SR Ca^2+^ leak, and promotes delayed afterdepolarizations [236,237,238,239]. ROS-mediated RyR2 modification (S-nitrosylation and carbonylation), together with SERCA2a inhibition, diminishes SR Ca^2+^ uptake and increases spontaneous SR Ca^2+^ release [202,240]. By destabilizing ΔΨm and promoting opening of the mitochondrial permeability transition pore (mPTP), mtROS disrupts ER–mitochondria contact sites and perturbs Ca^2+^ microdomain signaling [241,242]. At the ionic current level, ROS enhances late Na^+^ current (I_Na,L) by inducing S-nitrosylation of Nav1.5 and CaMKII-dependent phosphorylation. Augmentation of I_Na,L lengthens action potential duration (APD) and increases susceptibility to early afterdepolarizations (EADs) [243,244,245,246]. In parallel, oxidative stress suppresses repolarizing K^+^ currents (I_Kr [hERG], I_Ks, and I_to) in several models, potentially diminishing repolarization reserve [247,248]. Upregulated Na^+^/Ca^2+^ exchange (NCX) produces a depolarizing inward current from SR Ca^2+^ leak, facilitating delayed afterdepolarizations [249,250]. Gap-junctional coupling deteriorates as connexin-43 (Cx43) undergoes redox-dependent phosphorylation and accelerated ubiquitin-mediated turnover, leading to slowed conduction and increased conduction heterogeneity [251,252,253,254]. Autophagy maintains channel and scaffold quality control (for example, RyR2, Nav1.5, and Cx43) and preserves membrane microdomain organization, while selective removal of depolarized mitochondria limits mtROS and stabilizes ER–mitochondria interfaces [255,256]. Lipophagy limits the accumulation of triglyceride and ceramide, thereby preserving membrane properties and ion channel gating [257,258]. Defective autophagy and mitophagy sustain oxidized protein and dysfunctional mitochondria in ventricular arrhythmia [255,259,260]. Under β-adrenergic stimulation and during reperfusion, rapid increases in NOX2/NOX4- and mitochondria-dependent ROS accelerate these processes [261,262]. AMPK activation (exercise and metformin), mitochondria-targeted agents (such as MitoTEMPO or elamipretide), inhibition of CaMKII oxidation, suppression of late Na^+^ current (ranolazine), RyR2 stabilization (dantrolene), and autophagy stimulation (rapamycin, spermidine) have been shown to reduce mtROS, restore channel quality control, preserve gap-junctional coupling, and support excitation–contraction stability and rhythm [260,263,264]. Atrial fibrillation exhibits reduced autophagy signatures with Cx43 lateralization and conduction heterogeneity [254]. Restoration of autophagic flux improves channel quality control and reduces substrate complexity for reentry in preclinical models. Oxidants also modulate inward-rectifier potassium current (I_K1) and hyperpolarization-activated current (I-f), thereby destabilizing the resting potential, increasing atrial automaticity, and expanding the arrhythmogenic substrate under stress [229,248]. Therapeutically, arrhythmic substrate is reduced by suppressing late sodium current (for example, ranolazine), stabilizing RyR2 or limiting CaMKII oxidation, and augmenting mitophagy and autophagy to restore channel turnover and gap-junctional coupling.

### 3.6. Cardiac Aging

Cardiac aging is marked by a coordinated decline in autophagy–lysosome function, mitochondrial quality control, and Ca^2+^ homeostasis. The aged myocardium exhibits impaired lysosomal acidification, reduced TFEB and TFE3 activity, accumulation of lipofuscin and incompletely cleared autophagosomes, and progressive mtDNA damage, together leading to diminished autophagic flux and mitophagy [265,266]. During aging, AMPK activity diminishes while mTORC1 activity increases. In parallel, SIRT1/SIRT3 signaling diminishes, and NRF2 target gene expression decreases, impairing proteostasis, antioxidant defenses, and organelle quality control [267,268,269]. Mitochondrial dynamics shift toward DRP1-dependent fission with loss of MFN1, MFN2, and OPA1; cristae architecture is remodeled; and ER–mitochondria contact sites are perturbed, promoting Ca^2+^ dysregulation and increased susceptibility to permeability transition [266]. Proteostasis deteriorates as CASA through BAG3–HSPB8 and CMA via LAMP2 decline, and ubiquitin–proteasome function is attenuated, which compromises myofibrillar protein quality control and maintenance of sarcomere integrity [270]. Extracellular matrix remodeling involves increased collagen deposition and cross-linking, accumulation of AGE modifications, and shifts in titin isoform composition with reduced titin phosphorylation. These changes increase passive stiffness and impair diastolic relaxation [271]. Senescence-associated secretory signaling (SASP) and innate immune pathways, including NLRP3 and cGAS–STING activated by mtDNA leakage, promote chronic inflammation [272,273]. Endothelial dysfunction and microvascular rarefaction reduce perfusion and exacerbate oxidative stress [266,274]. Electrical remodeling is characterized by Cx43 alterations, fibrosis, and dispersion of repolarization, thereby increasing vulnerability to atrial and ventricular arrhythmias [254]. Evidence from preclinical aging models indicates that endurance training and structured exercise activate AMPK and improve lysosomal function; caloric restriction and time-restricted feeding suppress mTORC1 and upregulate ULK1 signaling; and small molecules such as spermidine and trehalose enhance autophagy and improve diastolic function while limiting fibrosis [275,276,277]. Additional strategies under investigation include mTOR inhibition, AMPK activation with metformin, and NAD^+^ repletion with nicotinamide riboside or NMN to activate SIRT1/SIRT3. Other approaches involve pharmacologic TFEB activation, lysosome-directed methods that enhance v-ATPase activity and TRPML1-dependent Ca^2+^ release, and mitophagy enhancers that strengthen PINK1/Parkin and BNIP3/NIX pathways [260,278,279]. Reduced autophagy and mitophagy link aging to bioenergetic inefficiency, contractile and electrical remodeling, and diastolic dysfunction, thereby highlighting actionable targets for cardioprotection [266]. Single-cell analyses of aged hearts reveal coordinated downregulation of lysosome–autophagy genes in cardiomyocytes and endothelial cells with parallel activation of innate immune pathways [280]. SASP maintains the pathological condition, whereas enhancing mitophagy reverses aspects of the phenotype in vivo [131]. Sex differences modulate autophagy and mitochondrial signaling, with estrogen receptor pathways supporting SIRT3 and antioxidant programs. Sex-based differences in autophagy and mitochondrial quality control may contribute to HFpEF susceptibility and aging-related diastolic remodeling and should inform sex-stratified dosing and analyses in trials of autophagy-directed therapies [281,282]. Consequently, cardioprotection in aging should leverage endurance training and caloric restriction or their mimetics, together with mTOR inhibition or AMPK and SIRT1/SIRT3 activation to restore TFEB-lysosome function and mitophagy, using sex-dependent dosing strategies.

### 3.7. Inflammation, Innate Immunity, and Noninfectious Injury

Mitochondrial dysfunction generates mtROS and releases mtDNA into the cytosol. Mitochondrial DAMPs (damage-associated molecular patterns) and pathogen-associated molecular patterns (PAMPs) activate NLRP3, Toll-like receptors (TLRs), RIG-I-like receptors, and the cGAS–STING pathway [283,284]. PAMP recognition initiates TLR signaling through the adaptor proteins MyD88 and TIR-containing adapter-inducing interferon-β (TRIF) [285]. Cytosolic viral RNA sensor retinoic acid-inducible gene I (RIG-I) and melanoma differentiation-associated protein 5 (MDA5) activate the mitochondrial adaptor mitochondrial antiviral signaling (MAVS), which recruits TANK-binding kinase 1/IκB kinase ε (TBK1/IKKε) to phosphorylate interferon regulatory factor 3 (IRF3) [286]. Cytosolic DNA is detected by cGAS, which generates cGAMP to activate STING and TBK1, leading to IRF3 activation [287]. These pathways induce type I interferons and pro-inflammatory cytokines and activate NLRP3. Autophagy and mitophagy limit DAMP generation by eliminating depolarized mitochondria, degrading oxidized mitochondrial constituents, and sustaining lysosomal function, thereby reducing caspase-1 activation, IL-1β/IL-18 maturation, and type I interferon signaling [288]. Autophagy targets inflammasome components for lysosomal degradation and clears ASC (apoptosis-associated speck-like protein containing a CARD; PYCARD) specks, thereby limiting pyroptosis [289]. In macrophages, autophagy facilitates apoptotic cell clearance (efferocytosis) and lipid handling, suppresses excessive NF-κB signaling, and promotes pro-resolving programs during tissue repair [290]. In neutrophils, autophagy regulates neutrophil extracellular trap release (NETosis), limiting microvascular obstruction and immunothrombosis in reperfused or pressure-overloaded myocardium [291]. In platelets, PINK1/Parkin-dependent mitochondrial quality control attenuates mitochondrial activation, lowers ROS levels, and reduces hyperreactivity [292]. In endothelial cells, autophagy maintains barrier function and NO signaling while restricting complement deposition and leukocyte adhesion, thereby limiting vascular inflammation [147,159]. In cardiac fibroblasts, autophagy limits myofibroblast activation, modulates TGF-β/SMAD signaling, and promotes controlled matrix turnover [293]. Lysosomal dysfunction impairs the clearance of inflammasome complexes and delays inflammation [294]. Disordered lipid handling elevates signaling lipids such as ceramides, diacylglycerols, and lysophosphatidylcholines that activate innate sensors and propagate chemokine and cytokine release [295,296,297]. In preclinical models, pharmacologic and physiologic strategies that activate AMPK, inhibit mTORC1, or enhance TFEB-dependent lysosomal biogenesis increase autophagic flux and improve degradative capacity [298]. cGAS–STING signaling occurs at ER and mitochondria–ER contact sites (MAMs) and is modulated by autophagy [299]. Mitochondria–ER contact sites function as hubs that couple Ca^2+^ microdomains and NOX-derived ROS to PINK1/Parkin-mediated mitophagy, LC3-associated membrane formation, and innate immune signaling, thereby modulating the transition from adaptive repair to chronic inflammation [300,301,302]. LC3-associated phagocytosis (LAP) and LC3-associated endocytosis (LANDO) promote the clearance of apoptotic cells and regulate inflammatory receptor trafficking, thereby limiting cytokine amplification in injured myocardia [303,304,305,306]. Autophagy also interacts with the PAMP pathway, while xenophagy and virophagy target intracellular pathogens and viral components. Furthermore, autophagic turnover of adaptor proteins modulates receptor signaling. Trained immunity programs in myeloid cells rely on mitochondrial metabolism, linking metabolic memory to cardiac inflammation [307,308]. Hence, combining modulation of the NLRP3 and IL-1 pathway with reinforcement of autophagy and mitophagy (AMPK activation, mTORC1 inhibition, TFEB-driven lysosomal biogenesis, LAP, and LANDO) attenuates caspase-1 activation and cytokine maturation, limits pyroptosis, and restores cytoprotective clearance of damaged mitochondria and assembled inflammasomes.

### 3.8. Fibroblast Activation and Matrix Remodeling

Cardiac fibroblasts integrate redox signaling with autophagy and lysosomal function to regulate myofibroblast transition and extracellular matrix turnover [309,310]. Mitochondrial reactive oxygen species and impaired autophagy promote TGF-β and SMAD-driven activation, enhance collagen synthesis and cross-linking, and reduce matrix resolution, which together increase stiffness and diastolic dysfunction [311,312]. Autophagy in fibroblasts limits inflammasome signaling and supports controlled matrix turnover, whereas lysosomal dysfunction prolongs inflammatory signaling and fibrosis [288,313]. Therapeutically, enhancement of autophagic flux and modulation of TGF-β signaling represent tractable anti-fibrotic strategies for clinical evaluation.

### 3.9. Therapeutic Modulation and Clinical Translation

Effective modulation of autophagy and redox pathways requires alignment with disease stage, cell type, and treatment duration rather than uniform activation or suppression [314]. A practical framework focuses on acute cytoprotection in ischemia–reperfusion and longer-term remodeling in cardiometabolic disease [179,315]. Structured exercise, metformin, and fasting regimens activate the AMPK/ULK1 axis and suppress mTORC1 [316,317,318]. In preclinical models and early-phase clinical studies, these approaches enhance autophagic flux, improve lysosomal function, and support mitochondrial quality control [319,320]. Enhancing TFEB activity and lysosomal biogenesis rescues defective autophagic flux and results in productive clearance [218]. Stabilization of v-ATPase function and augmentation of TRPML1-dependent lysosomal Ca^2+^ release show consistent effects in preclinical models [199,321]. Mitochondria-directed therapeutics, including mitochondria-accumulating antioxidants (MitoQ and SkQ derivatives) and inner-membrane stabilizers (elamipretide), reduce mitochondrial oxidative stress, preserve ΔΨm, and facilitate mitophagy in preclinical and early-phase clinical studies [322]. Small molecules, such as spermidine, resveratrol, and melatonin, activate SIRT pathways, PGC-1α, ULK1, and TFEB to promote mitochondrial turnover and limit inflammatory signaling [323,324,325]. Safety considerations should inform dosing and scheduling decisions [326]. Therapeutic scheduling and dosing should be tailored to disease stage and cellular context. During I/R, brief induction that supports lysosomal function and mitophagy is appropriate, whereas in chronic cardiometabolic disease, intermittent low-intensity activation of TFEB-driven lysosomal pathways and PINK1/Parkin-mediated mitophagy is preferred to preserve proteostasis, with lineage-specific priorities across cardiomyocytes, endothelium, fibroblasts, and myeloid cells. Excessive or sustained activation of autophagy depletes contractile proteins, induces lysosomal membrane permeabilization with cathepsin release, and triggers Na^+^–K^+^-ATPase-dependent cell death under severe stress [266,327,328]. Cell-type specificity is critical. Cardiomyocytes, endothelial cells, vascular smooth muscle cells, and immune cells exhibit distinct autophagic activity and divergent responses to identical stimuli [314]. Restricting drug exposure optimizes the efficacy–safety balance [315,329]. Important obstacles to clinical application remain. First, validated tissue-specific biomarkers of autophagy or mitophagy in humans are lacking, and circulating LC3 and p62 measures have limited sensitivity, specificity, and tissue resolution and are sensitive to pre-analytical variables, such as hemolysis and platelet activation [330,331]. Development of standardized extracellular vesicle assays for EV-derived LC3 or p62 fragments, mitophagy-linked peptide measurements, and clinically compatible imaging probes would enable real-time pharmacodynamic monitoring [332]. Second, optimal timing of therapy is not well defined, including immediate post-reperfusion versus subacute or chronic phases, and the duration and frequency of dosing for remodeling indications require systematic evaluation [315]. Third, rational multi-target strategies require formal trials that integrate metabolic control, organelle quality control, and inflammation with prespecified endpoints [333]. Enrichment strategies are likely to accelerate progress from preclinical signals to durable clinical benefit [206,322]. Clinical translation benefits from objective response criteria. Candidate biomarker panels integrate extracellular EV-derived LC3 and p62 fragments with circulating mitochondrial DNA and oxylipid and cardiac magnetic resonance parameters, such as extracellular volume fraction and T1 mapping, together with echocardiographic myocardial strain and ambulatory arrhythmia frequency and duration [334,335,336,337,338]. Cardiac-targeted delivery concentrates therapeutic agents in the myocardium and minimizes off-target exposure. Peptide-guided nanoparticles, cardiac homing sequences, and AAV9-based approaches are being evaluated as delivery platforms for pathway-directed therapeutics targeting AMPK, TFEB, and PINK1/Parkin signaling [339,340,341,342]. Pharmacodynamic confirmation in patients will require biomarker panels integrating extracellular vesicle markers, oxidized lipid markers, and non-invasive imaging [332,336]. To support clinical translation, we outline a fit-for-purpose framework that emphasizes analytical validity, biological specificity, pharmacodynamic sensitivity, and clinical utility. The core criteria are rigorous preanalytical control and predefined reproducibility, orthogonal validation against tissue or imaging readouts and human pathway perturbation, temporal responsiveness to target modulation, incremental value beyond established markers and cardiac magnetic resonance metrics, and feasibility for multicenter implementation with standardized materials and reporting. In practice, pathway-matched agents that tune AMPK–ULK1, TFEB–lysosome, and PINK1/Parkin signaling should be paired with cardiac-targeted delivery and adaptive, biomarker-guided dosing to translate these mechanisms into durable clinical benefit.

## 4. Future Research Directions

Future research should develop clinically validated in vivo measures of autophagic flux and mitophagy with reference standards, calibration to tissue and imaging endpoints, and defined preanalytical controls. Cell-type-resolved biomarker panels that combine extracellular vesicle LC3 or p62 fragments with cell-specific markers and circulating mitochondrial DNA are needed to quantify pathway activity across cardiomyocytes, endothelium, fibroblasts, and myeloid cells. Human-compatible imaging probes for lysosomal function and mitophagy should be advanced, prioritizing reporters of lysosomal acidity, cathepsin activity, and PINK1 and Parkin activity and benchmarking them against pharmacologic perturbation. Target selectivity for mitophagy modulation should be improved by comparing PINK1 and Parkin activators with receptor-based strategies that use BNIP3, NIX, and FUNDC1 and by defining off-target profiles at mitochondria–ER contact sites. Treatment timing across disease stages should be defined, with brief induction during ischemia and early reperfusion and periodic low-level induction in chronic disease, with dose and intervals guided by pharmacodynamic thresholds that avoid sustained LC3 accumulation or loss of contractile proteins. Finally, clinical trials should adopt standardized endpoints that integrate extracellular vesicle markers, circulating mitochondrial DNA, oxylipidomics, cardiac magnetic resonance T1 mapping, and strain and ambulatory arrhythmia metrics using shared reagents and common data elements.

## 5. Concluding Remarks

In cardiovascular pathologies, mtROS induce organelle injury and impair autophagy and mitophagy, leading to loss of cellular quality control. In cardiomyocytes, endothelial cells, vascular smooth muscle cells, and immune cells, mtROS-associated organelle injury with impaired autophagy and mitophagy links bioenergetic inefficiency to Ca^2+^ dysregulation, ion channel remodeling, innate immune activation, and fibroblast-driven matrix deposition. Cellular architecture determines whether Ca^2+^/redox signals preserve contractile and vascular function or promote arrhythmia, stiffening, and fibrosis. Mechanism-directed modulation of AMPK/ULK1, PINK1/Parkin, TFEB-driven lysosomal biogenesis, and NRF2-regulated antioxidant defenses improves organelle turnover, preserves ΔΨm, restores metabolic flexibility, and attenuates inflammatory signaling. Clinical translation requires tissue-specific biomarkers of autophagy and dosing and delivery matched to disease stage. Biomarker-guided combinations that rebalance metabolic flexibility, lysosomal function, and autophagy support cardiovascular benefits. Pathways of clinical relevance that are under investigation or suitable for evaluation in clinical trials include AMPK activation and mTOR modulation, enhancement of PINK1/Parkin-mediated mitophagy and TFEB-driven lysosomal biogenesis, inhibition of NLRP3 and interleukin 1, metabolic reprogramming with SGLT2 inhibitors, mitochondria-targeted therapeutics, and select microRNA-directed strategies.

## Figures and Tables

**Table 1:** Key terms and measurement approaches.

Term	Operational Definition	Representative Measurement
Autophagic flux	Net turnover of autophagic cargo from autophagosome formation to lysosomal degradation	LC3-II turnover with lysosomal inhibitors such as bafilomycin A1 or chloroquine, p62 or SQSTM1 degradation, tandem mRFP GFP LC3 reporter, electron microscopy quantifying autophagosomes and autolysosomes
Mitophagy	Selective autophagic clearance of mitochondria	PINK1 stabilization and Parkin recruitment, mt-Keima or mito QC reporters, LC3 colocalization with mitochondrial markers, loss of mitochondrial proteins during flux blockade
Lysosomal competence	Capacity of lysosomes to acidify and degrade cargo	Lysosomal pH probes, such as LysoSensor or LysoTracker, with calibration; cathepsin activity assays; DQ BSA degradation; TFEB nuclear localization as an indirect marker
TFEB activation	Induction of lysosome and autophagy gene programs by TFEB	Nuclear TFEB localization, expression of CLEAR network targets, reporter assays
Mitochondrial ROS (mtROS)	Reactive oxygen species generated within mitochondria	MitoSOX with appropriate controls, targeted redox probes such as roGFP Orp1, electron paramagnetic resonance when available
Mitochondrial membrane potential (ΔΨm)	Electrical potential across the inner mitochondrial membrane	TMRM or TMRE in non-quench mode with calibration, JC 1 with caution
Autophagosome–lysosome fusion	Fusion of LC3-positive autophagosomes with LAMP1- or LAMP2-positive lysosomes	STX17 SNAP29 VAMP8 assays, LC3 and LAMP co localization, tandem mRFP GFP LC3 quench analysis
Lysosomal membrane permeabilization	Loss of lysosomal integrity with cathepsin release	Galectin 3 puncta, acridine orange relocation, cathepsin activity in cytosol

## Data Availability

No new data were created or analyzed in this study.

## Abbreviations

•OH	Hydroxyl radical
^1^O_2_	Singlet oxygen
ABCA1	ATP-binding cassette transporter A1
ABCG1	ATP-binding cassette transporter G1
AGEs	Advanced glycation end products
AMBRA1	Autophagy and beclin-1-regulated autophagy protein 1
AMPK	AMP-activated protein kinase
APD	Action potential duration
ASC	Apoptosis-associated speck-like protein containing a CARD
ATGs	Autophagy-related genes
ATP	Adenosine triphosphate
BAG3	BCL2-associated athanogene 3
BCL-2	B cell lymphoma 2
BNIP3	BCL2/adenovirus E1B 19 kDa-interacting protein 3
CaMKII	Ca^2+^/calmodulin-dependent protein kinase II
CASA	Chaperone-assisted selective autophagy
CMA	Chaperone-mediated autophagy
DAMPs	Damage-associated molecular patterns
DFCP1	Double FYVE domain-containing protein 1
DNMT3A	DNA (cytosine-5)-methyltransferase 3A
DRP1	Dynamin-related protein 1
EADs	Early afterdepolarizations
eNOS	Endothelial NO synthase
ER	Endoplasmic reticulum
ESCRT	Endosomal sorting complexes required for transport
FIP200	Focal adhesion kinase family-interacting protein of 200 kDa
FUNDC1	FUN14 domain-containing 1
GABARAP	Gamma-aminobutyric acid receptor-associated protein
GPx	Glutathione peroxidase
GR	Glutathione reductase
GSH	Glutathione
GSSG	Glutathione disulfide
HOO•	Hydroperoxyl radical
HOPS	Homotypic fusion and vacuole protein sorting
I_Na,L	Late Na^+^ current
I/R	Ischemia–reperfusion
IDH	Isocitrate dehydrogenase
IRS	Insulin receptor substrate
KEAP1	Kelch-like ECH-associated protein 1
KLF	Krüppel-like factor
LANDO	LC3-associated endocytosis
LAMPf2	Lysosome-associated membrane protein 2
LAP	LC3-associated phagocytosis
LC3	Microtubule-associated protein light chain 3
MCU	Mitochondrial calcium uniporter
MDH	Malate dehydrogenase
mETC	Mitochondrial electron transport chain
MFN	Mitofusin
MPT	Mitochondrial permeability transition
mPTP	Mitochondrial permeability transition pore
mtDNA	Mitochondrial DNA
mTORC1	Mechanistic target of rapamycin complex 1
mtROS	Mitochondrial ROS
N_2_O_3_	Dinitrogen trioxide
NBR1	Neighbor of BRCA1 gene 1
NDP52	Nuclear dot protein 52 kDa
NETosis	Neutrophil extracellular trap release
NIX	BCL2/adenovirus E1B 19 kDa protein-interacting protein 3-like
NLRP3	NLR family pyrin domain-containing 3
NNT	Nicotinamide nucleotide transhydrogenase
NO	Nitric oxide
NOX	NADPH oxidase
NRF2	Nuclear factor erythroid 2-related factor 2
O_2_•^−^	Superoxide anions
ONOO^−^	Peroxynitrite
OPA1	Optic atrophy 1
OPTN	Optineurin
p62	Sequestosome 1
PGC-1α	Peroxisome proliferator-activated receptor gamma coactivator 1-alpha
PINK1	PTEN-induced kinase 1
PKC	Protein kinase C
PPARα	Peroxisome proliferator-activated receptor alpha
Prx	Peroxiredoxin
RAPTOR	Regulatory-associated protein of mTOR
RET	Reverse electron transport
ROS	Reactive oxygen species
Rubicon	RUN domain- and cysteine-rich domain-containing beclin-1-interacting protein
RyR2	Ryanodine receptor 2
SERCA2a	Sarcoplasmic reticulum Ca^2+^ATPase 2a
SGLT2	Sodium–glucose cotransporter-2
SIRT	Sirtuin
SNARE	Soluble N-ethylmaleimide-sensitive factor attachment protein receptor
SODs	Superoxide dismutases
SOD1	Cu/ZnSOD
SOD2	MnSOD
SOX9	SRY-box transcription factor 9
STX17	Syntaxin 17
TET2	Tet methylcytosine dioxygenase 2
TFEB	Transcription factor EB
TGF-β	Transforming growth factor beta
TLRs	Toll-like receptors
TRPML1	Transient receptor potential mucolipin 1
Trx2	Thioredoxin 2
TrxR	Thioredoxin reductase
ULK1	Uncoordinated-51-like kinase 1
UVRAG	UV radiation resistance-associated gene
VAMP8	Vesicle-associated membrane protein 8
WIPI2	WD repeat domain phosphoinositide-interacting protein 2
ZDHHC13	Zinc finger DHHC-type palmitoyltransferase 13
ΔΨm	Mitochondrial membrane potential
